# EGR1 Regulation of Vasculogenic Mimicry in the MDA-MB-231 Triple-Negative Breast Cancer Cell Line through the Upregulation of KLF4 Expression

**DOI:** 10.3390/ijms241814375

**Published:** 2023-09-21

**Authors:** Euitaek Jung, Young Han Lee, Sukjin Ou, Tae Yoon Kim, Soon Young Shin

**Affiliations:** 1 Department of Biological Sciences, Sanghuh College of Life Science, Konkuk University, Seoul 05029, Republic of Korea; mylife4sci@konkuk.ac.kr (E.J.); yhlee58@konkuk.ac.kr (Y.H.L.); o1207sj@konkuk.ac.kr (S.O.); yun0614@konkuk.ac.kr (T.Y.K.); 2 Cancer and Metabolism Institute, Konkuk University, Seoul 05029, Republic of Korea

**Keywords:** Early Growth Response 1, Kruppel-like factor 4, MDA-MB-231 cells, mitogen-activated protein kinase pathway, vasculogenic mimicry

## Abstract

Vasculogenic mimicry (VM) is an intriguing phenomenon observed in tumor masses, in which cancer cells organize themselves into capillary-like channels that closely resemble the structure and function of blood vessels. Although VM is believed to contribute to alternative tumor vascularization, the detailed regulatory mechanisms controlling these cellular processes remain poorly understood. Our study aimed to investigate the role of Early Growth Response 1 (EGR1) in regulating VM in aggressive cancer cells, specifically MDA-MB-231 triple-negative breast cancer cells. Our study revealed that EGR1 promotes the formation of capillary-like tubes by MDA-MB-231 cells in a 3-dimensional Matrigel matrix. EGR1 was observed to upregulate Kruppel-like factor 4 (KLF4) expression, which regulates the formation of the capillary-like tube structure. Additionally, our findings highlight the involvement of the ERK1/2 and p38 mitogen-activated protein kinase pathways in mediating the expression of EGR1 and KLF4, underscoring their crucial role in VM in MDA-MB-231 cells. Understanding these regulatory mechanisms will provide valuable insights into potential therapeutic targets for preventing VM during the treatment of triple-negative breast cancer.

## 1. Introduction

Cancer is a disease in which normal cells transform into malignant cells capable of uncontrolled proliferation, invasion of surrounding tissues, and metastasis to other organs [1]. Angiogenesis, the sprouting of new blood vessels from pre-existing vessels, is essential in classical tumor growth and metastasis models. Although successful efficacy has been observed in anti-angiogenic therapies, resistance to these drugs continues to be reported in certain tumor cases [2].

Vasculogenic mimicry (VM) is a phenomenon observed within tumor masses, where cancer cells organize themselves into capillary-like channels that closely resemble the structure and function of blood vessels [3]. The concept of VM was first proposed in uveal melanoma in 1999 [4] and has been shown to occur in various types of cancers, including glioblastoma [5], lung cancer [6,7], and breast cancer [8]. The channels formed by VM are not composed of endothelial cells but consist of cancer cells themselves. They exhibit networks rich with glycoproteins in the extracellular matrix and lack endothelial cell markers, such as CD31 and CD34. Therefore, the commonly used detection method for VM is CD31-Periodic acid-Schiff (PAS) double staining, and in this staining method, the CD31^−^/PAS^+^ channels are considered VM [9]. Additionally, in vitro-formed VM channels have been observed to possess perfusable lumens, as demonstrated by the injection of fluorescent dyes and scanning electron microscopy methods [3].

Although the molecular mechanisms underlying VM are poorly understood, the epithelial-to-mesenchymal transition (EMT) is believed to play a crucial role [10]. Transcription factors, such as SNAIL1/2, TWIST, and ZEB1/2, involved in the EMT have also been implicated in VM formation [11]. Moreover, the invasion and migration abilities acquired during EMT are critical cellular processes in angiogenesis [12]. Therefore, there appears to be a significant association between VM and EMT biology, suggesting that VM can also be considered a marker of tumor cell plasticity [13].

The clinical significance of VM has been reported previously. For example, small-cell lung cancer studies have shown that patients with high VM formation have lower survival rates [6]. In renal carcinoma, incorporating VM channel density into the calculation of the traditional microvessel density definition based on endothelial-dependent blood vessels correlates more strongly with cancer prognosis [14]. Furthermore, in aggressive melanoma, VM formation has been reported to be unaffected by angiogenesis inhibitors, suggesting it is one of the mechanisms underlying resistance to anti-angiogenic therapy [15].

Early growth response-1 (EGR1) is a transcription factor with a three-tandem C_2_H_2_ zinc finger domain at the C-terminus that binds to GC-rich sequences and regulates gene expression in response to various cellular stimuli, including growth factors, cytokines, and cellular stresses such as hypoxia [16]. When these signals stimulate cells, members of the mitogen-activated protein kinase (MAPK) family, including ERK1/2, p38, and JNK1/2, are phosphorylated and activate the ELK1 and SRF transcription factors [17]. Activated transcription factors bind to the EGR1 promoter and induce its rapid expression. Rapid expression of EGR1 proteins regulates various target genes containing the EGR1-binding sequence (EBS), which is primarily involved in the immune response, differentiation, cell proliferation, and survival [17]. EGR1 also participates in angiogenesis by regulating the expression of various pro-angiogenic factors in tumor cells [18,19] and mediates mesenchymal stem cell (MSC) differentiation into tendons [20]. In addition, we have demonstrated that EGR1 is crucial for the transdifferentiation of MSCs into endothelial cells [21].

Although VM is considered to contribute to alternative tumor vascularization, the detailed molecular mechanisms underlying these cellular processes remain to be elucidated. This study aimed to investigate whether EGR1 can regulate VM in aggressive cancer cells and to identify EGR1-target genes and signaling pathways.

## 2. Results

### 2.1. Vasculogenic Mimicry of Aggressive Breast Cancer Cells

**Figure 1:**
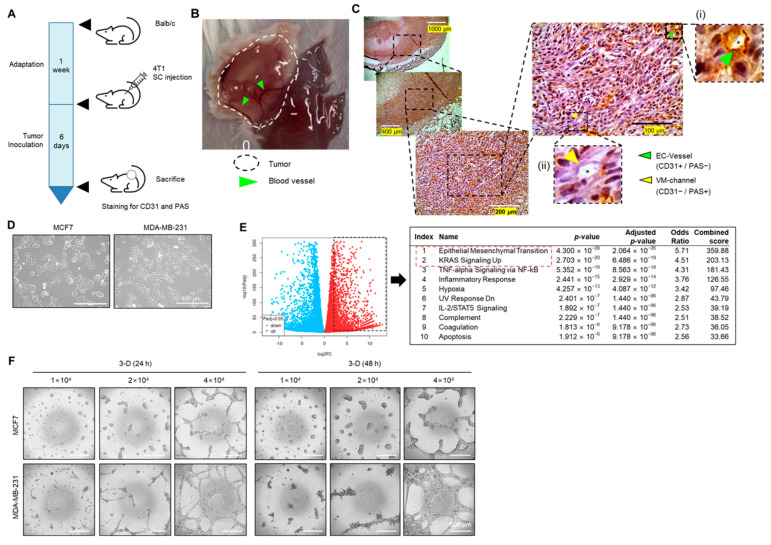
Formation of vasculogenic mimicry (VM) in MDA-MB-231 cells. (**A**) Experimental schedule for subcutaneous transplantation of 4T1 mouse mammary tumor cells in Balb/c mice. (**B**) Representative image of vascularized tumor mass after 6 days of tumor inoculation. The dotted line indicates the tumor region and the green arrowhead blood vessels. (**C**) Tumor sections were stained with Periodic acid–Schiff (PAS) staining (pink) and immunostained with anti-CD31 antibody (brown). Fixed and sectioned tumor masses displayed (**i**) CD31^+^/PAS^−^ endothelial cell (EC)-derived blood vessels and (**ii**) CD31^−^/PAS^+^ vasculogenic mimicry (VM) channels. *, lumen of VM channel (yellow arrowhead) or EC vessel (green arrowhead). (**D**) Morphology of MCF7 and MDA-MB-231 cells. (**E**) Transcriptome analysis and Enrichr tool results for upregulated genes in MCF7 and MDA-MB-231 cells. (**F**) Formation of VM in MCF7 cells and MDA-MB-231 cells observed through 3-dimensional (3-D) Matrigel culture, with images captured at 24 and 48 h.

We first examined whether VM occurs in the early stages of tumor development using a preliminary in vivo syngeneic model using 4T1 mouse mammary cancer cells. Mice were sacrificed at 4, 6, 8, and 10 days following the engraftment of 4T1 tumor cells. Tissue staining was conducted using both anti-CD31 antibody (an endothelial marker) and Periodic acid–Schiff (PAS) staining (a VM marker). We observed that on day 4, the transplanted tumor cells had not yet established a tumor mass. However, by day 8, a noticeable increase in blood vessel development was evident, indicated by the robust staining of anti-CD31 antibodies. These observations suggested that VM formation of cancer cells might occur before the angiogenesis of surrounding blood vessels. Based on these findings, we focused on the tissue samples collected on day 6 (Figure 1A). Tumor masses with vasculature were embedded in paraffin blocks (Figure 1B), and then CD31 and PAS double staining was performed. Results revealed the presence of a massive CD31^−^/PAS^+^ meshwork within the tumor mass. We observed both CD31+ endothelial channels and VM channels in actual luminal structures (Figure 1C, panel ii).

To confirm whether VM occurs predominantly in cancer cells with a mesenchymal phenotype due to EMT, we compared two breast cancer cell lines. The MCF7 cancer cell line exhibited an epithelial morphology, whereas the MDA-MB-231 cell line displayed a spindle-like mesenchymal morphology (Figure 1D). These phenotypic differences were also observed in the transcriptome analysis. By comparing the transcriptome data of the MDA-MB-231 and MCF7 cell lines obtained from a public database (GSE173986; [22]), significantly upregulated genes in the MDA-MB-231 cells were identified. Enrichment analysis revealed that the terms “epithelial-mesenchymal transition” and “KRAS signaling UP” were enriched in the upregulated genes, confirming that the MDA-MB-231 cells represent an aggressive and mesenchymal phenotype of breast cancer cells (Figure 1E).

For the in vitro assay of VM, a Matrigel-based tube formation assay was employed. The VM tube formation assay was performed with MCF7 and MDA-MB-231 cells in different densities, and it was observed that the MDA-MB-231 cells exhibited better VM formation at the same cell density (Figure 1F).

### 2.2. Tip-Cell-Like Behavior during VM Formation Was Observed in MDA-MB-231 Cells by Live Cell Imaging

**Figure 2:**
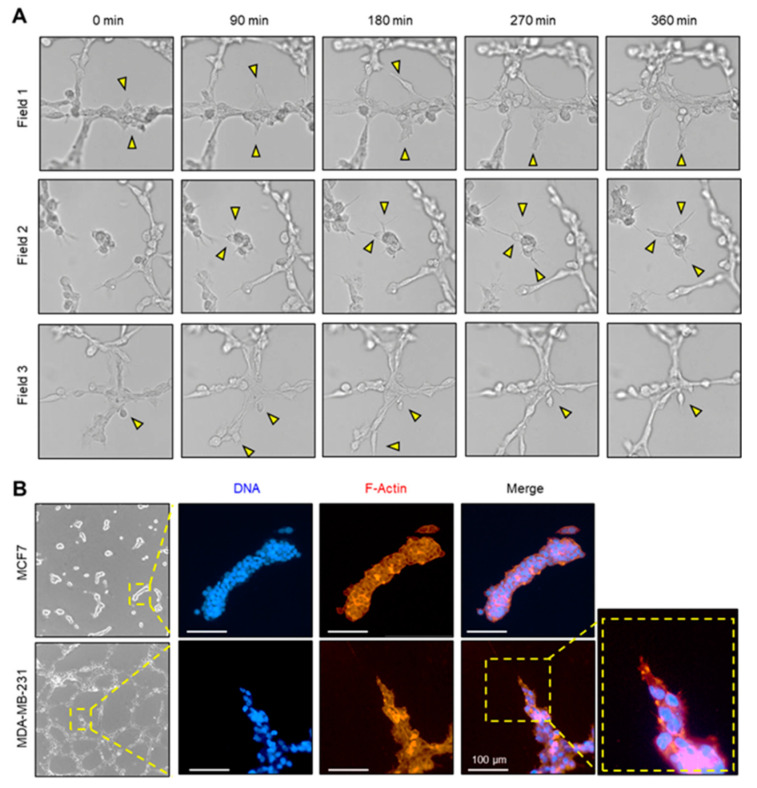
Tip cell-like behavior during vasculogenic mimicry (VM) formation of MDA-MB-231 cells. (**A**) MDA-MB-231 cells were seeded onto Matrigel, and VM structure was captured at 90-min intervals in three fields. Yellow arrowheads indicate areas of sprouting. The provided snapshots are extracted from Supplementary Videos S1–S3. (**B**) Whole mount staining of the VM model. MCF7 and MDA-MB-231 cells were seeded on Matrigel-coated coverslips and stained with Phalloidin-Rhodamine Red X for F-actin and Hoechst 33258 for DNA. Scale bar, 100 μm.

After the VM assay, cells exhibiting tip-cell-like morphological features, such as sprouting states and highly developed filopodia, were observed within the tube-like structures of MDA-MB-231 cells. Live-cell microscopy was used to observe this behavior. Three independent fields focused on regions where sprouting was expected were selected, and time-lapse images were captured. The results revealed that cells with well-developed filopodia within partially aggregated cell clusters at the edge of the VM structure sprouted towards the neighboring cells (Supplementary Videos S1–S3; Figure 2A). This behavior resembles the tip cell behavior of endothelial cells. Filopodia, which result from F-actin reorganization, were visualized using the F-actin-specific binding molecule phalloidin. A method for whole mount staining of the Matrigel model of VM formation was developed to facilitate this visualization. By applying this method to VMs formed by MCF7 and MDA-MB-231 cells, regions in the VMs of MDA-MB-231 cells that resembled sprouting angiogenesis were identified (Figure 2B).

### 2.3. EGR1 Positively Modulated the VM Formation

**Figure 3:**
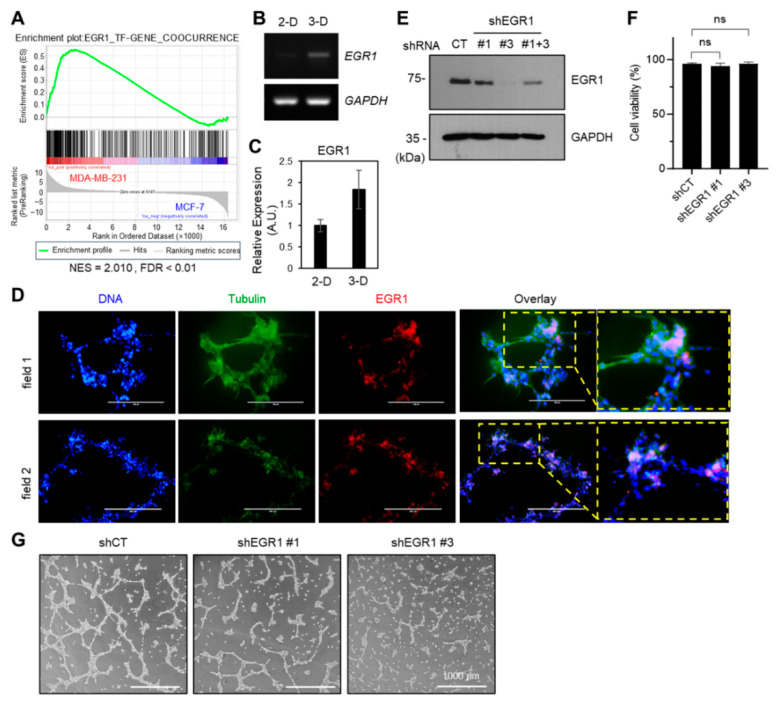
EGR1 regulates VM formation. (**A**) GSEA with gene set of EGR1 targets comparing transcriptome data of MCF7 and MDA-MB-231 cells. NES: Normalized enrichment score; FDR: False discovery rate q-value. (**B**) RT-PCR analysis of EGR1 mRNA expression in MDA-MB-231 cells cultured on 2D or 3D Matrigel-coated dishes. (**C**) The relative EGR1 expression levels were measured by real-time PCR. GAPDH level was used as the endogenous control. Error bars represent means ± S.D. (*n* = 3) (**D**) Whole-mount staining of VM structures using anti-EGR1 and anti-α/β-tubulin antibodies. Scale bar: 100 μm. (**E**) EGR1 expression levels were assessed in MDA-MB-231 cells expressing EGR1 shRNA (shEGR1) or control shRNA (shCT) through immunoblotting. # represents a distinct clone number. GAPDH was a loading control (shown in the left panels). (**F**) Cell viability for MDA-MB-231 transfectants was measured using the Cell Counting Kit-8. ns, not significant. (**G**) Representative images of VM structure in MDA-MB-231/shCT and shEGR1 #1 and #3 cells. Scale bar, 1000 μm.

Our previous study demonstrated that EGR1 is crucial for MSC differentiation into endothelial cells [21]. Furthermore, upon reanalyzing the transcriptome data of the MCF7 and MDA-MB-231 cell lines, the set of EGR1 transcription factor target genes was found to be enriched in the differentially upregulated genes in MDA-MB-231 cells (Figure 3A). Therefore, to investigate the involvement of EGR1 in the VM formation of cancer cells, we examined EGR1 expression during VM formation. RT-PCR and qPCR analyses show that EGR1 was upregulated in cells cultured in a 3D environment on Matrigel compared to 2D-cultured cells (Figure 3B,C). Furthermore, immunofluorescence staining revealed that EGR1 was localized in the nucleus of cells at the tube branch points (Figure 3D). Nuclear staining of EGR1 was also observed in the endothelial tip-like cells at the apex of a growing sprout (Supplementary Figure S1). These findings suggest that EGR1 plays a role in VM formation.

To investigate whether EGR1 is involved in VM formation in MDA-MB-231, we established stable transfectants expressing shRNAs targeting EGR1 (shEGR1 #1 and #3) and control transfectants expressing scrambled control shRNA (shCT) (Figure 3E). We observed that EGR1 knockdown did not significantly affect cell viability compared to shCT cells (Figure 3F). Upon conducting a tube formation assay using these transfectants, a noticeable decrease in VM formation was observed in shEGR1 transfectants (shEGR1 #1 and #3) compared to the shCT cells (Figure 3G). This finding suggests that EGR1 is involved in the process of VM formation.

### 2.4. KLF4, a Yamanaka Factor, Positively Regulates the VM Formation of Aggressive Breast Cancer Cell

**Figure 4:**
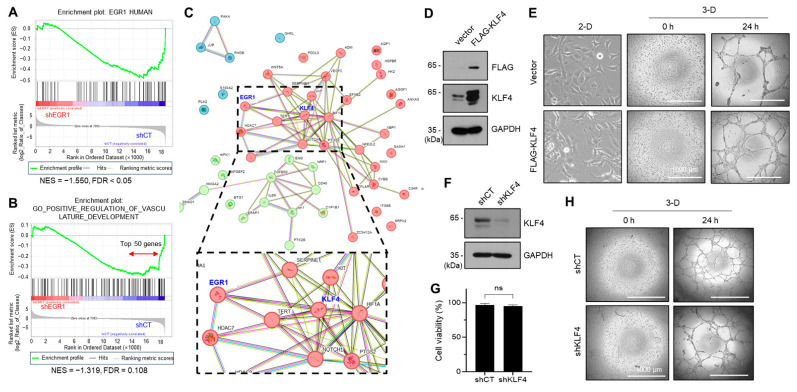
KLF4 positively regulates vasculogenic mimicry formation in MDA-MB-431 cells. (**A**,**B**) GSEA comparing transcriptomes of MDA-MB-231/shCT cells and MDA-MB-231/shEGR1 cells (**A**) and a gene set related to vasculature development regulation (**B**). NES: Normalized enrichment score; FDR: False discovery rate q-value. (**C**) The top 50 genes downregulated in shEGR1 cells indicated as a double arrow in (**B**) were analyzed for protein-protein interaction networks using the STRING database. (**D**) MDA-MB-231 cells were transfected with expression vectors for FLAG-KLF4 or empty vector (left panels). After cell lysis, immunoblotting was conducted using anti-FLAG and KLF4 antibodies. GAPDH was used as an internal control. (**E**) Morphology of MDA-MB-231 transfectants cultured in conventional culture plates (2-D). VM structure was imaged at 0 and 24 h after seeding to Matrigel-coated culture plates (3-D). Scale bar: 1000 μm. (**F**) MDA-MB-231 cells expressing control scrambled shRNA (shCT) or KLF4 shRNA (shKLF4) were lysed, and performed immunoblotting using anti-KLF4 antibodies GAPDH was used as an internal control. (**G**) Cell viability for shCT and shKLF4 cells was measured using a Cell Counting Kit-8. ns, not significant. (**H**) Representative images of VM structure in MDA-MB-231/shCT and shKLF4 cells. Scale bar, 1000 μm.

To elucidate the involvement of the EGR1 gene in VM formation, we conducted transcriptome analysis; we analyzed transcriptome data obtained from MDA-MB-231 transfectants expressing shCT and shEGR1 (accession number: GSE240489). From the results from GSEA, we verified the reduced expression of EGR1 target genes in shEGR1 cells compared to shCT cells (Figure 4A). Further analysis revealed that the gene set associated with the term “positive regulation of vasculature development” was downregulated in shEGR1 cells compared to the shCT cells (Figure 4B). The top 50 genes from this gene set were selected (Figure 4B and Supplementary Table S1) and analyzed for protein-protein interaction networks using the STRING database (Figure 4C). These findings suggest that KLF4, as the EGR1-regulated gene, could be a potential hub transcription factor in regulating endothelial differentiation of MDA-MB-231 cells. 

The KLF4 protein is an important transcription factor in embryonic stem cells and is one of the key factors in the generation of induced pluripotent stem cells (iPSCs), a term coined by Dr. Yamanaka [23,24]. The ‘Yamanaka factors’ OCT3/4, SOX2, KLF4, and c-MYC (OSKM) are essential for maintaining the characteristics of cancer stem cells [25,26]. KLF4 has also been shown to play a role in the transdifferentiation of head and neck cancer cell lines into endothelial cells [27].

To determine the contribution of KLF4 in VM formation, we transfected MDA-MB-231 cells with an expression vector for FLAG-tagged KLF4 (FLG-KLF4) or an empty vector (Figure 4D). VM formation appeared markedly increased in FLAG-KLF4 transfectants compared to empty vector-transfected cells (Figure 4E). To further corroborate the role of KLF4 in VM formation, we established stable transfectants expressing KLF4 shRNA (shKLF4) and control shRNA (shCT) (Figure 4F). We ascertained that KLF4 knockdown had no substantial impact on cell viability compared to the shCT cells (Figure 4G). VM formation was reduced in shKLF4 cells compared to the shCT cells, but complete elimination did not occur (Figure 4H), suggesting that KLF4 is necessary but not entirely sufficient for VM formation. These findings may underscore the role of KLF4 in the VM formation in MDA-MB-231 cells. 

### 2.5. EGR1 Regulates KLF4 Expression

**Figure 5:**
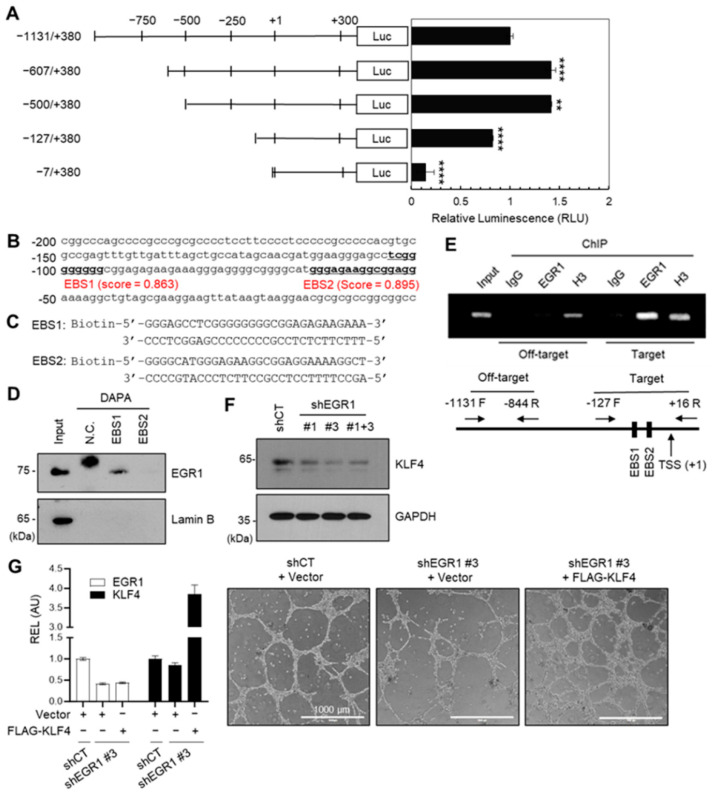
EGR1 regulates KLF4 expression. (**A**) Luciferase activities were measured in MDA-MB-231 cells transfected with a series of 5′-deletion constructs of human KLF4 promoter-luciferase plasmids (pKLF4-Luc). Error bars represent means ± S.D. (*n* = 3). ** *p* < 0.01 and **** *p* < 0.0001 compared to the pKLF4(−1131/+380) group using one-way ANOVA followed by Tukey’s multiple comparison test. (**B**) Genomic sequence of human KLF4 promoter (−1000/−1) showing putative EGR1-binding sequences (EBS1, EBS2) with calculated scores from JASPAR. (**C**) DAPA probes for EBS1 and EBS2 in KLF4 promoter. (**D**) DAPA and immunoblotting using nuclear extract of MDA-MB-231 cells. N.C.: negative control (no probe); Lamin B used as negative control. (**E**) ChIP assay of KLF4 promoter using an anti-EGR1 antibody, with PCR performed using site-specific primers. (**F**) Immunoblotting of shCT, shEGR1 #1, shEGR1 #3, and #1 + 3 cell lines using anti-EGR1 antibody. GAPDH was used as a loading control. (**G**) MDA-MB-231/shCT and MDA-MB-231/shEGR1 #3 cells were transfected with empty vector or FLAG-KLF4 expression vector and then seeded on Matrigel-coated plates. After 24 h, the VM structure was imaged (right panels). Scale bar, 1000 μm. The expression levels of EGR1 and KLF4 were determined using quantitative real-time PCR (left graph). Relative mRNA expression was represented by mean ± S.E.M. (*n* = 3). REL, relative expression level; AU, arbitrary unit.

After confirming that KLF4 mediates VM in aggressive breast cancer cells, we investigated the regulatory mechanism of human KLF4 expression. The human KLF4 promoter region was cloned from −1131 to +380 and serially deleted, followed by subcloning into a luciferase reporter vector (pGL4.17). These constructs (pKLF4-Luc) were transfected into MDA-MB-231 cells, and luciferase activity was measured to screen for the minimal promoter region. The promoter activity significantly decreased when the −127/+1 region was deleted, indicating that this region served as the minimal core promoter (Figure 5A).

Subsequently, the −127/+1 region was analyzed to determine whether the EGR1 transcription factor could bind to the minimal promoter. Two putative EGR1-binding sequences (EBS1, EBS2) were identified (Figure 5B). Furthermore, the public ChIP-Seq databases (ENCODE, ReMap) confirmed that EGR1 binds to promoter regions, including EBS1 and EBS2 (Supplementary Figure S2). Remarkably, the ChIP-Seq peaks of p300 and TBS were also co-localized with the EGR1 peak.

To investigate the binding of EGR1 to putative EBS1 and EBS2 sites within the −127/+1 region, we designed biotin-labeled probes specific for EBS1 and EBS2 (Figure 5C) and conducted a DNA affinity precipitation assay (DAPA) using nuclear lysates extracted from MDA-MB-231 cells (Figure 5D). The DAPA analysis demonstrated that EGR1 was associated exclusively with the EBS1 site. To determine whether EGR1 binds to the EBS1 site at the chromatin level, we performed a chromatin immunoprecipitation (ChIP) assay employing an anti-EGR1 antibody in MDA-MB-231 cells. Remarkably, our ChIP analysis revealed a distinct binding of EGR1 to the EBS1 site within the −127/+1 region of the KLF4 promoter (Figure 5E). 

To further corroborate the role of EGR1 in KLF4 expression, we examined the KLF4 levels in MDA-MB-231/shCT and shEGR1 #1 and #3 cells. We observed a notable reduction in KLF4 protein expression in shEGR1 cells compared to shCT cells, affirming the regulatory role of EGR1 in KLF4 expression (Figure 5F). To determine the role of KLF4 in EGR1-induced VM formation, we introduced FLAG-tagged KLF4 into shEGR1 #3 cells. The expression levels of transfected KLF4 were confirmed using quantitative real-time PCR (qPCR) analysis (Figure 5G, left graph). Under this experimental condition, the reduced VM formation due to EGR1 knockdown exhibited partial restoration upon introducing exogenous KLF4 expression (Figure 5G, right panels). These findings suggest that EGR1-regulated KLF4 could contribute to EGR1-induced VM formation.

### 2.6. ERK and p38 Kinases Regulate EGR1 and KLF4 Expression

**Figure 6:**
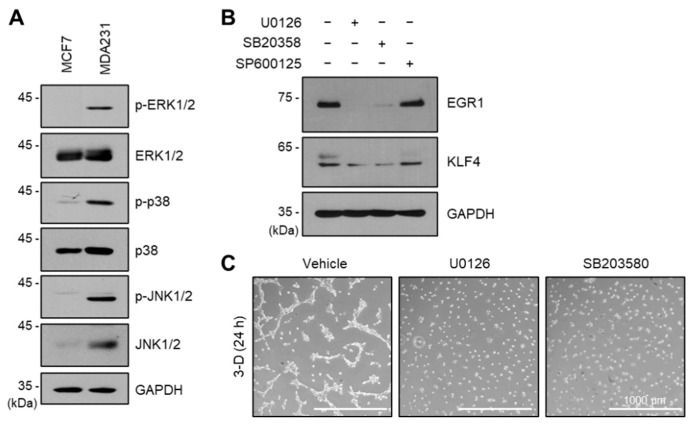
ERK and p38 regulate EGR1 and KLF4 expression. (**A**) Immunoblotting assay of serum-starved MCF7 and MDA-MB-231 cells using indicated antibodies. GAPDH was used as a loading control. (**B**) Immunoblotting was performed on MDA-MB-231 cells treated with 10 μM U0126, 20 μM SB203580, or 25 μM SP60012. (**C**) MDA-MB-231 cells were cultured in 3-dimensional (3-D) Matrigels in the presence or absence of 10 μM U0126 and 20 μM SB203580. After 24 h, images of the VM structure were captured. Scale bar: 1000 μm.

In a previous transcriptome analysis, the KRAS signaling pathway was upregulated in the aggressive cancer cell line MDA-MB-231 compared to that in the MCF7 cell line (Figure 1B). MDA-MB-231 cells also carry a constitutively active KRAS mutant (G13D) [28]. Since EGR1 is regulated by the downstream kinase MAPK family in the oncogenic KRAS signaling pathway [29,30], we investigated whether constitutively activated MAPK increased EGR1 expression. Indeed, when comparing serum-starved MCF7 and MDA-MB-231 cells, ERK1/2, p38, and JNK1/2, the three MAPK proteins were all phosphorylated in MDA-MB-231 cells (Figure 6A). To determine the role of MAPKs in the expression of EGR1 and KLF4, we employed chemical inhibitors targeting MAPKs. Our findings demonstrated that treatment with U0126 (MAPK Kinase inhibitor) or SB203580 (p38 inhibitor) resulted in a notable reduction in the protein levels of both EGR1 and KLF4, whereas treatment with SP600125 (JNK inhibitor) exhibited only a minor impact on their expression (Figure 6B). 

To determine whether ERK and p38 kinases regulate VM in MDA-MB-231 cells, we performed a tube formation assay using U0126 and SB203580 inhibitors. As predicted, VM was blocked entirely by both inhibitors (Figure 6C). These findings suggest that the ERK/p38-EGR1-KLF4 signaling pathway mediates VM in aggressive cancer cells.

### 2.7. Visualizing the Activation of the EGR1-KLF4 Signaling Pathway during VM Formation Using a Fluorescent-Based Reporter System

KLF4 is a critical transcription factor in embryonic stem cells and is important for CSC maintenance of cancer stem cells [23,25,26]. A previous study developed a reporter system capable of detecting the activation state of transcription factors to track cancer stem cell populations through live-cell imaging [31]. Based on this concept, we developed and employed a biosensor system sensing EGR1-regulated KLF4 promoter activity during VM formation. 

**Figure 7:**
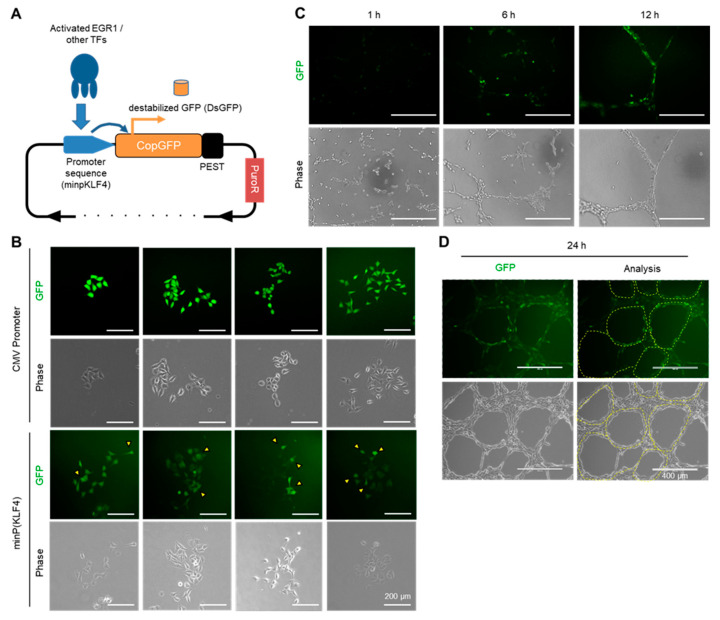
Visualizing the activation of the EGR1-KLF4 signaling pathway during VM formation using a fluorescent-based reporter system. (**A**) Vector map of the fluorescent-based reporter system showing regulation of destabilized GFP (dsGFP) expression by the minP(KLF4) sequence. PEST is a peptide sequence characterized by a high content of proline, glutamic acid, serine, and threonine. (**B**) Activity diversity of minP(KLF4) within a single colony. MDA-MB-231/CMV-dsGFP or minP(KLF4)-dsGFP cells were seeded at low density to allow for the formation of single colonies. After 4 days, established single colonies were imaged. Yellow arrowheads indicate the more “active” cells. Scale bar: 200 μm. (**C**) Activation of minP(KLF4) during VM formation. MDA-MB-231/minP(KLF4)-dsGFP cells were seeded onto Matrigel and imaged after 1 h, 6 h, and 12 h. Scale bar: 400 μm. (**D**) Activity diversity of minP(KLF4) within VM tube structures. MDA-MB-231/minP(KLF4)-dsGFP cells were seeded onto Matrigel-coated coverslips. Images were taken after 24 h. The yellow dashed line in the “Analysis” panel indicates the border of the “edge” of the tubes. Scale bar: 400 μm.

First, we subcloned the minimal promoter region responsible for KLF4 expression, named ‘minP(KLF4)’, corresponding to the −127/−1 sequence. This minP(KLF4) region was inserted into a promoter region of a vector encoding copGFP with the PEST sequence (destabilized GFP, named dsGFP). The PEST sequence increases protein turnover and minimizes background GFP expression. The minP(KLF4) sequence allows GFP to be selectively expressed only in cells where the transcription factors involved in KLF4 expression, including EGR1, are activated (Figure 7A). 

MDA-MB-231 cells were transfected with either this reporter vector (minP(KLF4)) or a vector controlled by a constitutively activated CMV promoter and subjected to puromycin selection. These transfectants were seeded at a low density to allow colony formation from a single cell. As a result, while the CMV promoter exhibited uniform GFP signals in all cells within a colony, using minP(KLF4) revealed varying GFP signals among cells within every colony (Figure 7B). This finding suggests that the EGR1-KLF4 signaling pathway is activated only in response to unknown factors. Furthermore, VM formation assays were observed using the minP(KLF4)-dsGFP cell line and GFP signals. The GFP signal increased following VM formation after seeding (Figure 7C). Moreover, GFP activation was predominantly observed at the edges of the VM tubes, where VM sprouting and tip cell behavior occurred (Figure 7D). These data suggest that the EGR1-KLF4 signaling pathway plays a significant role in VM formation and VM tip cell-like behavior.

In summary, activation of ERK and p38 leads to upregulation of EGR1, which subsequently activates the expression of the KLF4 gene. The expressed KLF4 protein positively regulates the formation of VM in triple-negative MDA-MB-231 breast cancer cells. 

## 3. Discussion

The tumor vasculature is classically considered to be formed by sprouting angiogenesis from pre-existing blood vessels surrounding the tumor [32]. However, recent findings have shown that anti-angiogenic therapy based on the classical tumor angiogenesis model is not fully effective [2], and the tumor vasculature has characteristics different from those of normal blood vessels [33]. In addition, various cell types exist within the tumor microenvironment [34], and some of these cells acquire an endothelial-like phenotype [15]. Therefore, in addition to the classical tumor vascularization mechanism through sprouting angiogenesis of pre-existing blood vessels surrounding the tumor mass, it is conceivable that tumor vascularization can occur more rapidly through an alternative mechanism in which stromal cells located near the tumor mass or cancer cells themselves within the tumor mass undergo a transition into endothelial-like cells.

Cancer cells have been reported to acquire an endothelial-like phenotype, which is referred to as VM [3,4]. The aggressiveness of cancer cells is often evaluated based on the extent of EMT, which has been reported to be highly associated with the occurrence of VM [10,35]. Consistently, by reanalyzing public transcriptome data and using a Matrigel-based tube formation assay, we demonstrated that MDA-MB-231 breast cancer cells exhibit more aggressive characteristics than MCF7 cells and are prone to VM (Figure 1). In addition, tip-cell-like behavior during VM formation was observed using live-cell imaging and whole mount staining (Figure 2). Although the detailed process and molecular mechanism of VM remain largely unexplored, it is noteworthy that the behavior of tip cells during sprouting angiogenesis can be interpreted as undergoing a mesenchymal transition [12], and there is a strong correlation between EMT and VM formation [10].

Furthermore, in this study, we observed tip-cell-like behavior in cancer cells during endothelial tube-like formation (Figure 2). This finding suggests that a subset of the highly aggressive population of cells undergoes a transition towards the tip-cell-like phenotype, closely resembling the process of sprouting angiogenesis, contributing to the induction of VM. Further investigation is needed to identify the signals that guide the sprouting of VM tip cells.

During VM, the EGR1 protein expression increased, which was confirmed by RT-PCR. Whole mount staining revealed that the EGR1 protein was located in the nuclei of tip-cell-like VM cells (Figure 3). In addition, the knockdown of EGR1 resulted in the failure of VM, indicating that EGR1 primarily participates in VM (Figure 3). EGR1 has also been reported to regulate EMT [17,36,37], and the findings of this study further demonstrate that EGR1 is a regulator of VM.

Public transcriptome data also predicted that KLF4 is upregulated during VM (Figure 4). Overexpression and knockdown experiments revealed that KLF4 is a positive regulator of VM. In addition, promoter analysis of KLF4 and further investigations showed that KLF4 is an EGR1 target gene (Figure 5). The EGR1-KLF4 signaling pathway is regulated by ERK and p38 kinases (Figure 6), which are likely activated by the oncogenic mutant KRAS (G13D) in MDA-MB-231 cells [28]. Various oncogenic driver mutations, including KRAS mutations, mediate the aggressiveness and EMT of cancer cells [1,38]. This study suggests that the oncogenic KRAS-ERK/p38-EGR1-KLF4 signaling axis is a potential regulatory signaling pathway that mediates VM in aggressive breast cancer cells.

KLF4, a member of the Yamanaka factor family (OCT3/4, SOX2, KLF4, and c-MYC, collectively referred to as OSKM), is known to play a crucial role in embryonic stem cells and is also implicated in maintaining stemness and EMT processes in the context of cancer stem cell models [23,25,26]. From the perspective of interpreting VM as the differentiation of cancer stem cells into endothelial-like cells [39,40], it can be inferred that EGR1 maintains the stemness and endothelial differentiation capacity of cancer stem cells by regulating KLF4. Different promoter activities among individual cells, even within a single colony, were observed using the biosensor system dsGFP under the regulation of the minP(KLF4) (−127/−1 bp upstream). This can be attributed to asymmetric division or differentiation of cancer stem cells. Furthermore, it was confirmed that minP(KLF4) is activated during VM formation and exhibits predominant activity at the edges of VM tubular structures, where VM tip cells can emerge (Figure 7). These data suggest that the EGR1-KLF4 pathway is activated in the VM-forming subpopulation of cells derived from cancer stem cells.

## 4. Materials and Methods

### 4.1. Reagents and Plasmids

U0126, SB203580, and SP600125 were purchased from Calbiochem (San Diego, CA, USA) and were dissolved in dimethyl sulfoxide. The full-length open reading frame encoding human KLF4 was subcloned into the pCMV-Tag4B vector (Stratagene, La Jolla, CA, USA) to generate a C-terminal FLAG tag. 

### 4.2. Cell Culture

Human breast cancer cell lines (MCF7 and MDA-MB-231) and a mouse mammary carcinoma cell line (4T1) were obtained from the American Type Culture Collection (ATCC, Manassas, VA, USA) and cultured in Dulbecco’s modified DMEM medium supplemented with 10% fetal bovine serum and 1% penicillin-streptomycin (BioWest, Kansas City, MO, USA).

### 4.3. Animal Study

BALB/c mice (male, 6–8 weeks old) were purchased from Orient Bio, Inc. (Seongnam, Republic of Korea). 4T1 cells (5 × 10^5^ cells/mouse) were subcutaneously injected into the mice (*n* = 5). After six days, the mice were sacrificed by exposure to CO_2_, and the tumor tissues were fixed in 3.7% paraformaldehyde overnight at 25 °C. After embedding in paraffin blocks, tumor tissues were cut into 5 μm sections. For CD31-PAS staining, CD31 was probed using Vectastain Elite ABC-HRP Kit (Vector Laboratories, Burlingame, CA, USA). After the DAB reaction, sections were incubated in 0.5% (*w/v*) periodic acid for 10 min and stained with Schiff’s reagent (Sigma-Aldrich, St. Louis, MO, USA) for another 10 min. The nuclei were stained with hematoxylin, and the sections were mounted. 

### 4.4. VM Tube Formation Assay

For VM formation assay, 2 × 10^4^ cells of MCF7 or MDA-MB-231 were suspended in 100 μL of the culture media and seeded onto the Matrigel. Assays were performed in triplicate. After 12–24 h, the capillary-like network structure was imaged using an EVOS FL Auto microscope (Thermo Fisher Scientific, Waltham, MA, USA). For whole mount staining of the VM structure on Matrigel, the VM tube formation assay was performed on the coverslips. Briefly, 50 μL of Matrigel was smeared onto 18 mm round coverslips and placed in a 12-well plate. After 15 min at RT, the Matrigel-coated coverslips were solidified and submerged in culture media to prevent drying. MCF7 and MDA-MB-231 cells (2 × 10^5^ cells/well) were seeded in 12-well plates containing coverslips. After 24 h, the VM structures were fixed with 3.7% PFA for 20 min at RT without shaking. This fixation process causes problematic liquefication of Matrigel. This issue was resolved by immersing the coverslips in a 1× PBS/0.5% agarose solution at around 60 °C for 30 s, immobilizing the fixed VM structures. The resulting immobilized VM structures were subjected to an immunofluorescence staining procedure with increased incubation time to ensure antibody penetration. 

### 4.5. Transcriptome Analysis

Public transcriptome data (GSE173986; [22]) were obtained from the Gene Expression Omnibus (GEO) [41] and reanalyzed to obtain normalized data using GEO2R. Gene Set Enrichment Analysis (GSEA) was performed using the Enrichr tool for screening enriched terms (available at https://maayanlab.cloud/Enrichr, accessed on 23 April 2023) [42] and another GSEA software (http://www.broadinstitute.org/gsea/index.jsp, accessed on 23 April 2023) provided by the joint project of UC San Diego (San Diego, CA, USA) and Broad Institute of MIT and Harvard University [43]. Protein interactome analysis was performed using STRING software [44]. Reanalyzed transcriptome data and the gene sets used for GSEA are provided in Supplementary Data and Supplementary Table S2, respectively.

### 4.6. Reverse Transcription (RT)-PCR and Quantitative Real-Time PCR (qPCR)

Cells were lysed in NucleoZOL (Thermo Fisher Scientific, Waltham, MA, USA), and total RNA was extracted according to the manufacturer’s instructions. Equal amounts (1 μg) of RNA were used to synthesize cDNA using an iScript cDNA Synthesis Kit (Bio-Rad, Hercules, CA, USA). The cDNA samples were subjected to standard PCR using gene-specific primer pairs. The sequences of primer pairs were KLF4 Forward, 5′-TCT CAA GGC ACA CCT GCG AA-3′; KLF4 Reverse, 5′-GGT CGC ATT TTT GGC ACT GG-3′; EGR1 Forward, 5′-CAG CAG TCC CAT TTA CTC AG-3′; EGR1 Reverse, 5′-GAC TGG TAG CTG GTA TTG-3′; GAPDH Forward, 5′-ACC CAC TCC TCC ACC TTT G-3′; GAPDH Reverse, 5′-CTC TTG TGC TCT TGC TGG G-3′. The PCR conditions were as follows: 4 min at 95 °C for the initial denaturation, followed by 30 cycles of 30 s at 95 °C, 30 s at 60 °C, and 30 s at 72 °C. Amplicons were resolved on a 2% agarose gel and visualized under UV light. For qPCR, cDNA samples were amplified using AccuPower^®^ 2× GreenStar™ qPCR Master Mix (Bioneer, Daejeon, Korea), and real-time fluorescence was measured by the CFX Connect system (Bio-Rad). qPCR primer pairs were EGR1 Forward, 5′-AGT TTG CCA GGA GCG ATG AA-3′; EGR1 Reverse, 5′-GGG ACG GGT AGG AAG AGA GA-3′; GAPDH Forward, 5′-ACC CAC TCC TCC ACC TTT G-3′; GAPDH Reverse, 5′-CTC TTG TGC TCT TGC TGG G-3′. The relative expression levels of EGR1 were calculated by the 2^−ΔΔCt^ method, setting GAPDH as the endogenous control. The assay was performed in triplicate.

### 4.7. Generation of MDA-MB-231 Transfectants Expressing shRNA

For establishing knockdown of EGR1 or KLF4 in MDA-MB-231 cells, we inserted double-stranded oligonucleotide templates encoding short hairpin RNA (shRNA) into the pSilencer 2.1 U6 Neo vector (Thermo Fisher Scientific) by using HindIII and BamHI restriction enzyme. The target sequences of each gene were as follows: shCT (scrambled control), 5′-ACT ACC GTT GTT ATA GGT GT-3′; shEGR1 #1, 5′-GTT ACT ACC TCT TAT CCA T-3′; shEGR1 #3, 5′-CAT CTC TCT GAA CAA CGA GAA-3′; shKLF4, 5′-GGA CGG CTG TGG ATG GAA A-3′. The constructed expression vectors were introduced into MDA-MB-231 cells using Lipofectamine 2000 (Thermo Fisher Scientific), according to the manufacturer’s instructions.

### 4.8. Immunoblotting

Total cell lysates were extracted via RIPA buffer (50 mM Tris-HCl [pH 7.4], 400 mM NaCl, 1% Triton X-100, 0.25% sodium deoxycholate, 1 mM EDTA, 1 mM Na_3_VO_4_, and 1 mM NaF). Next, 15–30 μg of the lysates were resolved using SDS-PAGE in 7–10% gels and transferred to 0.45 µm nitrocellulose membranes. The membranes were incubated in TBS-T (10 mM Tris-HCl [pH 7.5], 150 mM NaCl, 0.01% Tween-20) with 5% skim milk and incubated in diluted primary antibodies overnight at 4 °C. The next day, the membranes were incubated with appropriate secondary antibodies for 1 h and visualized using a WestPico PLUS Chemiluminescent Substrate (Thermo Fisher Scientific). The antibodies used for immunoblotting are listed in Supplementary Table S3.

### 4.9. Cell Viability Assay

Cell viability was determined using a Cell Counting Kit-8 (CCK-8; Dojindo Molecular Technologies, Gaithersburg, MD, USA) according to the manufacturer’s instructions. Briefly, MDA-MB-231 transfectants expressing control shRNA (shCT), shEGR1, or shKLF4 were plated onto a 96-well culture plate at a density of 5 × 10^3^ cells/well in 100 μL of culture medium. The cells were then cultured for 24 h prior to adding a CCK-8 solution. Following 1 h, absorbances at 450 nm were measured using an Emax Endpoint ELISA Microplate Reader (Molecular Devices, Sunnyvale, CA, USA).

### 4.10. KLF4 Promoter Cloning and PROMOTER-Reporter Activity Assay

For cloning the KLF4 promoter region, we retrieved the genomic sequence for KLF4 (NM_004235.6) from the UCSC Genome Browser (https://genome.ucsc.edu/, accessed on 14 January 2022) and searched the Candidate Cis-Regulatory Elements (cCREs), histone modification mark (H3K27AC), which is often found near the regulatory elements of the KLF4 gene, TATA-box binding protein (TBP), p300 histone acetyltransferase, ChIP sequence-based transcription factor clusters, and EGR1 using Encyclopedia of DNA Elements (ENCODE) database (http://www.encodeproject.org/, accessed on 23 April 2023). We observed that EGR1 could be bound to near 500 bp from the transcription start site, and the ChIP-Seq peaks of p300 and TBP binding sequences were co-localized with the EGR1-binding peak (Supplementary Figure S2). Based on this analysis, the serial 5′-truncated fragments of the KLF4 promoter were amplified from human genomic DNA and cloned into the pGL4.17 reporter vector (Promega, Madison, WI, USA). The primers used to amplify the KLF4 promoter fragments were −1131F (5′-TGA GGT CAG GTG TTC GAG AC-3′), −609F (5′-GCG AGG AAC CGT GCG AG-3′), −500F (5′-CTG GAT GAG TCA CGC GGA TAA-3′), −127F (5′-CAT AGC AAC GAT GGA AGG GA-3′), +7F (5′-GGC GGC CGG CAG TTT C-3′), and +380R (5′-AGC AAG GCG AGT AAG TAG GTC-3′). The pKLF4-Luc construct was introduced into MDA-MB-231 cells. According to the manufacturer’s instructions, luciferase activity was measured using the Dual-Glo Luciferase Assay System (Promega).

### 4.11. DNA Affinity Precipitation Assay (DAPA)

MDA-MB-231 cells were harvested, and nuclear proteins were isolated using the Nuclear and Cytoplasmic Extraction Kit (Thermo Fisher Scientific), as described previously [21]. Briefly, cells were lysed in a hypotonic lysis buffer (Cytoplasmic Extraction Reagent I; CER I). After centrifugation at 15,000× *g* for 5 min, the pellet was resuspended in CERII reagent, centrifuged at 15,000× *g* for 10 min, and collected nuclear proteins in the supernatants.

The nuclear proteins (100 μg) were diluted in DNA binding buffer (10 mM HEPES-NaOH [pH 8.0], 50 mM KCl, 100 mM NaCl, 5 mM MgCl_2_, 0.1% NP-40, and 0.25 mM EDTA) and pre-incubated with 0.5 μg/mL of salmon sperm DNA at 4 °C. After 30 min, 4 μg of the biotinylated or unlabeled oligonucleotides were added, and the mixture was incubated for 2 h at 4 °C. The structures and sequences of DAPA probes are shown in Figure 5C. The DNA-protein complexes were pulled down using 30 μL of streptavidin-conjugated agarose resin (Cat. no.20353, Thermo Fisher Scientific). The beads were washed three times with the binding buffer and boiled in 2× Laemmli sample buffer. The supernatants were resolved using 8% PAGE and subjected to immunoblotting. 

### 4.12. Chromatin Immunoprecipitation (ChIP) Assay

MDA-MB-231 cells (4 × 10^6^ cells/sample) were cross-linked using 1% paraformaldehyde, and the ChIP assay was performed using a Magnetic ChIP Kit (Thermo Fisher Scientific) according to the manufacturer’s instructions. For each immunoprecipitation, 8 μg of the antibody was used. The ChIP samples were subjected to PCR using locus-specific primer pairs. The primer sequences for the target site were 5′-CAT AGC AAC GAT GGA AGG GA-3′ (−127F KLF4) and 5′-TGG TCG GGA AAC TGC CGG-3′ (+16R KLF4). Off-target primer pairs (−1131F KLF4, 5′-TGA GGT CAG GTG TTC GAG AC-3′; −844R KLF4, 5′-CGC GGA GAC AGT TTT CAA CC-3′) were used as negative controls.

### 4.13. The Fluorescent Reporter for KLF4 Promoter

The design of the destabilized copGFP (dsGFP) reporter vectors (pminP(KLF4)-dsGFP and pCMV-dsGFP) to trace the activity of the promoter sequence was based on a previous study [22]. The reporter vector was constructed by modifying the pRetroQ-acGFP-N1 vector (Clontech) in-house (a simplified vector map is shown in Figure 7A). The reporter vector was transfected into MDA-MB-231 cells and selected for MDA-MB-231/minP (KLF4)-dsGFP staining using 2 mg/mL puromycin.

### 4.14. Statistical Analysis

Data were analyzed using GraphPad Prism version 8.3.1 (GraphPad Software Inc., La Jolla, CA, USA) and expressed in terms of means ± S.D. Statistical analysis was performed using a Student’s *t*-test or one-way analysis of variance, followed by Tukey’s or Sidak’s multiple comparisons test. Differences with *p* < 0.05 were considered statistically significant.

## 5. Conclusions

Our findings demonstrate that EGR1 plays a crucial role in facilitating VM in aggressive cancer cells by regulating KLF4 expression. EGR1 regulated KLF4 and vasculogenic expression in aggressive MDA-MB-231 breast cancer cells. The induction of the EGR1 protein, mediated by ERK1/2 and p38 MAPKs during VM formation, activates the minimal promoter region (−127/−1) of the KLF4, which acts as a positive regulator of VM. Considering the stressful conditions within the tumor mass, such as hypoxia, that have been shown to promote the aggressiveness of cancer cells [45,46], the VM mechanism presented in this study is likely to significantly contribute to the alternative and rapid vascularization in the tumor mass. Consequently, targeting the VM process could be a promising therapeutic strategy for combating aggressive tumors in anticancer therapy.

## Data Availability

The data discussed in this publication have been deposited in NCBI Gene Expression Omnibus and are accessible through GEO Series accession number GSE240489 (https://www.ncbi.nlm.nih.gov/geo/query/acc.cgi?acc=GSE240489, released on 15 August 2023).

## Supplementary Materials

The following supporting information can be downloaded at: https://www.mdpi.com/article/10.3390/ijms241814375/s1.

## Abbreviations

ChIP	Chromatin immunoprecipitation
DAPA	DNA affinity precipitation assay
EGR1	Early Growth Response 1
EBS	EGR1-binding sequence
EMT	Epithelial-to-mesenchymal transition
GSEA	Gene Set Enrichment Analysis
GEO	Gene Expression Omnibus
KLF4	Kruppel-like factor 4
MAPK	Mitogen-activated protein kinase
MSC	Mesenchymal stem cell
PAS	Periodic acid-Schiff
RT-PCR	Reverse transcriptase-PCR
shRNA	Short hairpin RNA
VM	Vasculogenic mimicry
