# Supplementary material for: EGR1 Regulation of Vasculogenic Mimicry in the MDA-MB-231 Triple-Negative Breast Cancer Cell Line through the Upregulation of KLF4 Expression
Source: Int J Mol Sci. 2023 Sep 21;24(18):14375. doi: 10.3390/ijms241814375 (PMC10532327; doi:10.3390/ijms241814375)
Supplement: Supplementary file 1 [file ijms-24-14375-s001.zip › Supplementary Figure S1.pdf]

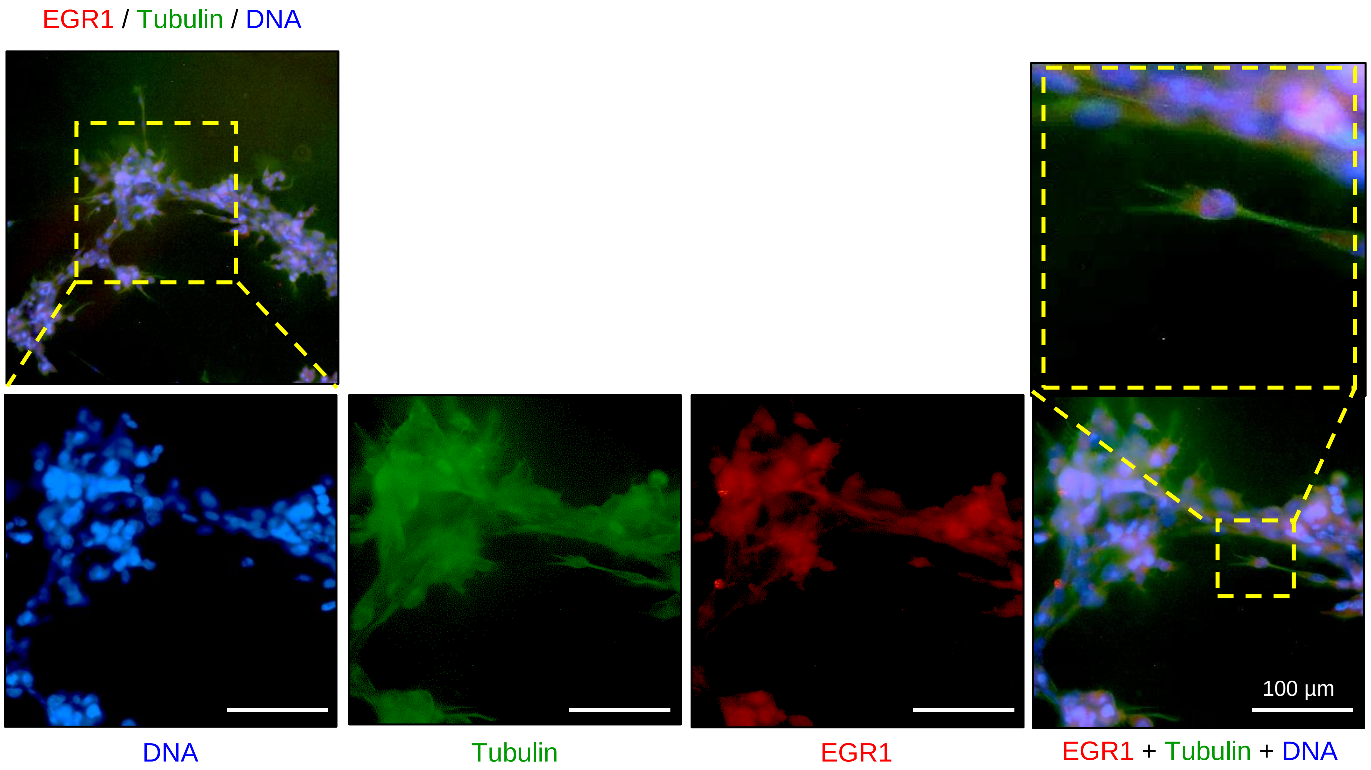

Supplementary Figure S1. Whole-mount staining of VM structures using anti-EGR1 and anti-α/β-tubulin antibodies. Scale bar: 100 µm. Nuclear staining of EGR1 was observed in the endothelial tip-like cells at the apex of a growing sprout (highlighted by the right dashed box).
