# Supplementary material for: EGR1 Regulation of Vasculogenic Mimicry in the MDA-MB-231 Triple-Negative Breast Cancer Cell Line through the Upregulation of KLF4 Expression
Source: Int J Mol Sci. 2023 Sep 21;24(18):14375. doi: 10.3390/ijms241814375 (PMC10532327; doi:10.3390/ijms241814375)
Supplement: Supplementary file 1 [file ijms-24-14375-s001.zip › Supplementary Figure S2.pdf]

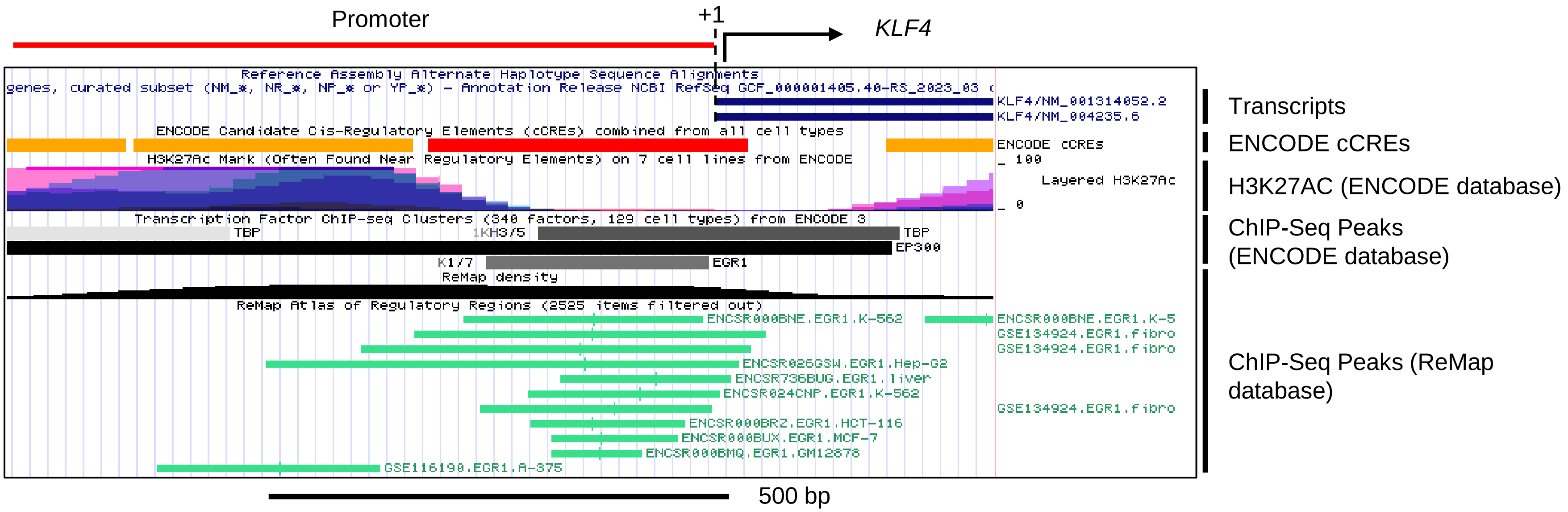

Supplementary Figure S2. Genomic landscape of human KLF4 promoter. '+1' is the transcription start site of KLF4. The figure was drawn in UCSC Genome Browser. Descriptions of each track are also denoted on the right. ChIP-Seq peaks of EGR1, p300, and TBP are used in ENCODE database, whereas EGR1 data is used in ReMap database. Scale bar, 500 bp.
