# Supplementary material for: EGR1 Regulation of Vasculogenic Mimicry in the MDA-MB-231 Triple-Negative Breast Cancer Cell Line through the Upregulation of KLF4 Expression
Source: Int J Mol Sci. 2023 Sep 21;24(18):14375. doi: 10.3390/ijms241814375 (PMC10532327; doi:10.3390/ijms241814375)
Supplement: Supplementary file 1 [file ijms-24-14375-s001.zip › Supplementary Table S1.pdf]

Supplementary Table S1.

Gene	Title	Score
**PTGS2**	prostaglandin-endoperoxide synthase 2 [Source:HGNC Symbol;Acc:HGNC:9605]	-4.798136
**S100A1**	S100 calcium binding protein A1 [Source:HGNC Symbol;Acc:HGNC:10486]	-4.791465
**SOD2**	superoxide dismutase 2 [Source:HGNC Symbol;Acc:HGNC:11180]	-4.043826
**WNT5A**	Wnt family member 5A [Source:HGNC Symbol;Acc:HGNC:12784]	-3.228846
**CYBB**	cytochrome b-245 beta chain [Source:HGNC Symbol;Acc:HGNC:2578]	-2.828556
**HK2**	hexokinase 2 [Source:HGNC Symbol;Acc:HGNC:4923]	-2.689266
**CFLAR**	CASP8 and FADD like apoptosis regulator [Source:HGNC Symbol;Acc:HGNC:1876]	-2.409688
**JAK1**	Janus kinase 1 [Source:HGNC Symbol;Acc:HGNC:6190]	-2.326097
**XBP1**	X-box binding protein 1 [Source:HGNC Symbol;Acc:HGNC:12801]	-2.254697
**IL6R**	interleukin 6 receptor [Source:HGNC Symbol;Acc:HGNC:6019]	-2.229708
**NOTCH1**	notch receptor 1 [Source:HGNC Symbol;Acc:HGNC:7881]	-2.19651
**ZC3H12A**	zinc finger CCCH-type containing 12A [Source:HGNC Symbol;Acc:HGNC:26259]	-2.18782
**AGGF1**	angiogenic factor with G-patch and FHA domains 1 [Source:HGNC Symbol;Acc:HGNC:24684]	-2.154218
**GHRL**	ghrelin and obestatin prepropeptide [Source:HGNC Symbol;Acc:HGNC:18129]	-1.703607
**ITGB8**	integrin subunit beta 8 [Source:HGNC Symbol;Acc:HGNC:6163]	-1.46311
**NRP1**	neuropilin 1 [Source:HGNC Symbol;Acc:HGNC:8004]	-1.338356
**KIT**	KIT proto-oncogene, receptor tyrosine kinase [Source:HGNC Symbol;Acc:HGNC:6342]	-1.287901
**PDCL3**	phosducin like 3 [Source:HGNC Symbol;Acc:HGNC:28860]	-1.278304
**HIPK1**	homeodomain interacting protein kinase 1 [Source:HGNC Symbol;Acc:HGNC:19006]	-1.249922
**SERPINE1**	serpin family E member 1 [Source:HGNC Symbol;Acc:HGNC:8583]	-1.219398
**RAPGEF2**	Rap guanine nucleotide exchange factor 2 [Source:HGNC Symbol;Acc:HGNC:16854]	-1.166402
**EGR1**	early growth response 1 [Source:HGNC Symbol;Acc:HGNC:3238]	-1.118414
**TGFBR2**	transforming growth factor beta receptor 2 [Source:HGNC Symbol;Acc:HGNC:11773]	-1.103925
**PAK4**	p21 (RAC1) activated kinase 4 [Source:HGNC Symbol;Acc:HGNC:16059]	-1.081508
**SRPX2**	sushi repeat containing protein X-linked 2 [Source:HGNC Symbol;Acc:HGNC:30668]	-1.060884
**SMAD1**	SMAD family member 1 [Source:HGNC Symbol;Acc:HGNC:6767]	-1.018937
**EFNB2**	ephrin B2 [Source:HGNC Symbol;Acc:HGNC:3227]	-1.005226
**C3AR1**	complement C3a receptor 1 [Source:HGNC Symbol;Acc:HGNC:1319]	-0.915103
**HDAC7**	histone deacetylase 7 [Source:HGNC Symbol;Acc:HGNC:14067]	-0.859571
**AQP1**	aquaporin 1 (Colton blood group) [Source:HGNC Symbol;Acc:HGNC:633]	-0.834743
**PTK2B**	protein tyrosine kinase 2 beta [Source:HGNC Symbol;Acc:HGNC:9612]	-0.797913
**KLF4**	KLF transcription factor 4 [Source:HGNC Symbol;Acc:HGNC:6348]	-0.78216
**ENG**	endoglin [Source:HGNC Symbol;Acc:HGNC:3349]	-0.755928
**HIF1A**	hypoxia inducible factor 1 subunit alpha [Source:HGNC Symbol;Acc:HGNC:4910]	-0.727883
**PLK2**	polo like kinase 2 [Source:HGNC Symbol;Acc:HGNC:19699]	-0.724163
**ERAP1**	endoplasmic reticulum aminopeptidase 1 [Source:HGNC Symbol;Acc:HGNC:18173]	-0.701251
**HDAC9**	histone deacetylase 9 [Source:HGNC Symbol;Acc:HGNC:14065]	-0.6565
**ADM**	adrenomedullin [Source:HGNC Symbol;Acc:HGNC:259]	-0.650301
**HSPB6**	heat shock protein family B (small) member 6 [Source:HGNC Symbol;Acc:HGNC:26511]	-0.633375
**HMGA2**	high mobility group AT-hook 2 [Source:HGNC Symbol;Acc:HGNC:5009]	-0.571515
**JUP**	junction plakoglobin [Source:HGNC Symbol;Acc:HGNC:6207]	-0.569813
**BTG1**	BTG anti-proliferation factor 1 [Source:HGNC Symbol;Acc:HGNC:1130]	-0.532886
**TERT**	telomerase reverse transcriptase [Source:HGNC Symbol;Acc:HGNC:11730]	-0.515895
**CD40**	CD40 molecule [Source:HGNC Symbol;Acc:HGNC:11919]	-0.504138
**SASH1**	SAM and SH3 domain containing 1 [Source:HGNC Symbol;Acc:HGNC:19182]	-0.503938
**CYP1B1**	cytochrome P450 family 1 subfamily B member 1 [Source:HGNC Symbol;Acc:HGNC:2597]	-0.464693
**RHOB**	ras homolog family member B [Source:HGNC Symbol;Acc:HGNC:668]	-0.460672
**NFE2L2**	NFE2 like bZIP transcription factor 2 [Source:HGNC Symbol;Acc:HGNC:7782]	-0.449504
**VEGFC**	vascular endothelial growth factor C [Source:HGNC Symbol;Acc:HGNC:12682]	-0.439679
**ANXA3**	annexin A3 [Source:HGNC Symbol;Acc:HGNC:541]	-0.401073
