# Supplementary material for: EGR1 Regulation of Vasculogenic Mimicry in the MDA-MB-231 Triple-Negative Breast Cancer Cell Line through the Upregulation of KLF4 Expression
Source: Int J Mol Sci. 2023 Sep 21;24(18):14375. doi: 10.3390/ijms241814375 (PMC10532327; doi:10.3390/ijms241814375)
Supplement: Supplementary file 1 [file ijms-24-14375-s001.zip › Supplementary Table S2.pdf]

Supplementary Table S2. Description of gene sets used in this study.

Gene set	Genes	Library	Description
EGR1 HUMAN	70	TRRUST Transcription Factors 2019	Database of human and mouse transcriptional regulatory networks by sentence-based text mining and manual curation.
EGR1 TF-GENE COOCURRENCE	284	Enrichr Submissions TF-Gene Coocurrence	Genes that most commonly co-occur with a transcription factor. This library was created from gene sets submitted to Enrichr by non-frequent users.
GO POSITIVE REGULATION OF VASCULATURE DEVELOPMENT	108	GO Biological Process 2023	Any process that activates or increases the frequency, rate or extent of vasculature development. (GO_REF:0000058)
