# Supplementary material for: EGR1 Regulation of Vasculogenic Mimicry in the MDA-MB-231 Triple-Negative Breast Cancer Cell Line through the Upregulation of KLF4 Expression
Source: Int J Mol Sci. 2023 Sep 21;24(18):14375. doi: 10.3390/ijms241814375 (PMC10532327; doi:10.3390/ijms241814375)
Supplement: Supplementary file 1 [file ijms-24-14375-s001.zip › Supplementary Table S3.pdf]

Supplementary Table S3. Antibodies used in this study.

Primary antibodies	Manufacturer	Cat. no.	Applications
CD31	Abcam	ab28364	IHC
EGR1	Cell Signaling Technology	4153	IB, IF, ChIP
p-ERK1/2 (T202/Y204)	Santa Cruz Biotechnology	sc-7383	IB
ERK2	Santa Cruz Biotechnology	sc-154	IB
p-p38 (T180/Y182)	Cell Signaling Technology	9211	IB
p38	Cell Signaling Technology	9212	IB
p-JNK1/2 (T183/Y185)	Santa Cruz Biotechnology	sc-6254	IB
JNK1	Santa Cruz Biotechnology	sc-571	IB
KLF4	Cell Signaling Technology	4038	IB
FLAG	Cell Signaling Technology	14793	IB
α/β-Tubulin	Invitrogen	ma1-19400	IF
GAPDH	Santa Cruz Biotechnology	sc-32233	IB
Lamin B	Santa Cruz Biotechnology	sc-6216	IB
Histone H3	Cell Signaling Technology	4620	ChIP

Secondary antibodies	Manufacturer	Cat. no.	Applications
anti-mouse IgG – HRP	Invitrogen	31430	IB
anti-rabbit IgG – HRP	Invitrogen	31460	IB
anti-goat IgG – HRP	Invitrogen	31402	IB
anti-mouse IgG – Alexa 488	Invitrogen	A-11001	IF
anti-rabbit IgG – Alexa 555	Invitrogen	A-21428	IF

Control antibodies	Manufacturer	Cat. no.	Applications
normal rabbit IgG	Cell Signaling Technology	2729	ChIP

IB, Immunoblotting; IF, Immunofluorescence staining, ChIP, Chromatin immunoprecipitation; IHC, Immunohistochemistry
